# The optimal sequence of bronchial brushing and washing for diagnosing peripheral lung cancer using non-guided flexible bronchoscopy

**DOI:** 10.1038/s41598-020-58010-w

**Published:** 2020-01-23

**Authors:** Jun Hyeok Lim, Min Jeong Kim, Sang-Hoon Jeon, Mi Hwa Park, Woo Youl Kim, Minkyung Lee, Jun Ho Kim, Jung Soo Kim, Young Sam Kim, Lucia Kim, Kyung-Hee Lee, Seung Min Kwak, Hyekyung Shin, Hae-Seong Nam

**Affiliations:** 1 Division of Pulmonary and Critical Care Medicine, Department of Internal Medicine, Inha University Hospital, Inha University School of Medicine, Incheon, 22332 Republic of Korea; 2 Department of Nuclear Medicine, Inha University Hospital, Inha University School of Medicine, Incheon, 22332 Republic of Korea; 3 Department of Radiology, Inha University Hospital, Inha University School of Medicine, Incheon, 22332 Republic of Korea; 4 Department of Thoracic Surgery, Inha University Hospital, Inha University School of Medicine, Incheon, 22332 Republic of Korea; 5 Department of Pathology, Inha University Hospital, Inha University School of Medicine, Incheon, 22332 Republic of Korea; 6 Division of Pulmonary and Critical Care Medicine, Department of Internal Medicine, Incheonsarang Hospital, Incheon, 22135 Republic of Korea

**Keywords:** Lung cancer, Lung cancer, Lung cancer, Lung cancer

## Abstract

The optimum sequence of bronchial brushing and washing for diagnosing peripheral lung cancer, defined as an invisible endobronchial tumour, is not clear and requires further study. We prospectively obtained washing samples after brushing in patients with peripheral lung tumours during non-guided flexible bronchoscopy (FB) to investigate the diagnostic yield of these samples and conducted a retrospective review of the prospectively collected data. The study included 166 patients who met the inclusion criteria. The overall diagnostic yield of bronchial brushing and washing for peripheral lung cancer was 52.4%. The diagnostic yields of brushing and washing were 37.3% and 46.4%, respectively, and that of washing was superior according to McNemar’s test (*p* = 0.017, κ = 0.570). Furthermore, washing was diagnostic, whereas brushing was not, in 15.1% of all cases. Comparison of positive washing cytology (brushing) with the respective pathological diagnosis yielded a concordance rate of 88.3% (90.3%), with κ = 0.769 (0.801) (*p* < 0.001). Performing washing after brushing during non-guided FB is a very safe, cost-effective procedure that may help improve the diagnostic yield in patients with suspected peripheral lung cancer. Our information will also benefit clinicians performing diagnostic bronchoscopy in patients with suspected peripheral lung cancer when fluoroscopic guidance or advanced bronchoscopy techniques are not available.

## Introduction

Flexible bronchoscopy (FB) is an essential step in the diagnosis of lung cancer. Numerous basic diagnostic procedures using FB, including bronchoalveolar lavage or washing, brushing, endobronchial or transbronchial biopsy (TBB), and transbronchial needle aspiration, have been evaluated in various combinations to improve the diagnostic yield of FB in patients with suspected lung cancer^1–3^. The diagnostic yield of bronchoscopy for lung cancer depends on whether the tumor is visible within the bronchial tree. The overall sensitivity of FB among many studies of central tumours, defined as visible endobronchial tumours, was 88% (range 67–97%). The sensitivities of endobronchial biopsy, brushing, and washing were 74% (range 48–97%), 61% (23–93%), and 47% (29–78%), respectively. In comparison, the overall sensitivity of FB for peripheral lung cancer, defined as an invisible endobronchial tumour, was 78% (range 36–88%). TBB had the highest sensitivity (57%, range 17–80%), followed by brushing (54%, range 16–84%), and bronchoalveolar lavage or washing (43%, range 12–65%)^3^. However, the majority of studies of peripheral lung cancer used fluoroscopic guidance routinely and also performed TBB. Fluoroscopy has the limitation of not being universally available, and TBB has a higher risk of pneumothorax compared with other FB procedures^1,4^.

Current advanced bronchoscopy techniques, such as electromagnetic navigation bronchoscopy, radial-probe endobronchial ultrasound (EBUS), ultrathin bronchoscopy, and virtual bronchoscopy, have facilitated biopsy of peripheral lung tumours. However, the diagnostic yield of these techniques for peripheral lung cancer does not meet expectations, despite improved navigation and imaging of peripheral lung tumours. Moreover, these advanced bronchoscopy techniques are expensive and not feasible in every institution^5,6^.

Consequently, advanced bronchoscopy techniques and fluoroscopy are not available in most institutions, and bronchial brushing and washing are safe, cost-effective diagnostic techniques for patients with peripheral lung tumours. However, the optimum sequence of brushing and washing samples for diagnosing peripheral lung cancer is not clear and requires further study^7^. Few studies have examined the sequence of brushing and washing without TBB in patients with peripheral lung tumours during non-guided FB.

Accordingly, we hypothesised that washing samples obtained after abrading the bronchial surface with a brush in the target bronchus will increase the amount of tumour cells collected and improve the diagnostic yield of peripheral lung cancer. To address this, we prospectively obtained washing samples after brushing in patients with peripheral lung tumours during non-guided FB to investigate the diagnostic yield of these samples and conducted a retrospective review of the prospectively collected data.

## Results

### Patient characteristics

The study included 166 patients who met the inclusion criteria. All consecutive eligible patients underwent other procedures for diagnosis of lung cancer after brushing and washing procedures of the FB, including CT-guided transthoracic needle biopsy (CT-TTNB, *n* = 102), video-assisted thoracic surgery (*n* = 14), pleural cytology or biopsy (*n* = 10), tissue cores via EBUS–transbronchial needle aspiration of a mediastinal lymph node (*n* = 31), and biopsy or aspiration of extrapulmonary lesions (*n* = 9). Some EBUS procedures were performed in the same bronchoscopy session after the FB, other procedures were performed at another time or on another day after the FB. The baseline characteristics of the study population are summarised in Table 1. The median age of the patients was 69 (range 31–88) years, and there were 122 (73.5%) males. The majority of the patients were former or current smokers (69.9%) and had a histology of adenocarcinoma (71.1%). Based on the chest CT findings before the FB procedure, the target tumour was located in the right upper (25.9%), middle (8.4%), and lower (25.3%) lobes and left upper (24.7%) and lower (5.7%) lobes. The mean ± standard deviation of the tumour size and SUV_max_ were 43.2 ± 21.4 (range 6–116) mm and 9.5 ± 4.9 (range 1.2–27.7), respectively. The final stage, including the clinical or pathological stage, was I in 22.3% of patients, II in 10.8%, III in 22.9%, and IV in 44.0%.

**Table 1:** Baseline patient characteristics according to the diagnostic yields of bronchial brushing and washing in patients with peripheral lung cancer during non-guided flexible bronchoscopy.

Variables	No. (%) *n* = 166	Diagnostic yield of brushing		Diagnostic yield of washing	
		No. (%)	*p* value	No. (%)	*p* value
Age			0.471		0.823
< 70	89 (53.6)	31 (34.8)		42 (47.2)	
≥ 70	77 (46.4)	31 (40.3)		35 (45.5)	
Gender			0.351		0.575
Men	122 (73.5)	43 (35.2)		55 (45.1)	
Women	44 (26.5)	19 (43.2)		22 (50.0)	
Smoking			0.643		0.948
Never	50 (30.1)	20 (40.0)		23 (46.0)	
Current + Former	116 (69.9)	42 (36.2)		54 (46.6)	
Final diagnosis			0.983		0.906
ADC	118 (71.1)	43 (36.4)		52 (44.1)	
SQC	32 (19.3)	15 (46.9)		20 (62.5)	
Others NSCC	8 (4.8)	0 (0)		1 (12.5)	
SCC	8 (4.8)	4 (50.0)		4 (50.0)	
Tumor size (mm)			<0.001		0.001
<30	50 (30.1)	7 (14.0)		12 (24.0)	
30 ≤ < 70	99 (59.6)	44 (44.4)		55 (55.6)	
≥70	17 (10.2)	11 (64.7)		10 (58.8)	
Location of tumor			0.133		0.158
BUL + RML	98 (59.0)	32 (32.7)		41 (41.8)	
BLL	68 (41.0)	30 (44.1)		36 (52.9)	
Bronchus sign			0.001		<0.001
Yes	93 (56.0)	45 (48.4)		58 (62.4)	
No	73 (44.0)	17 (23.3)		19 (26.0)	
SUV_max_ (*n* = 157)			0.082		0.026
<6.00	39 (24.8)	8 (20.5)		11 (28.2)	
6.00 ≤ < 12	73 (46.5)	33 (45.2)		39 (53.4)	
≥12.00	45 (28.7)	18 (40.0)		24 (53.3)	
Final stage			0.01		0.013
I–II	55 (33.1)	13 (23.6)		18 (32.7)	
III–IV	111 (66.9)	49 (44.1)		59 (53.2)	

ADC, adenocarcinoma; SQC, squamous cell carcinoma; NSCC, non-small cell carcinoma; SCC, small cell carcinoma; BUL, both upper lobe included right/left upper lobe; RML, right middle lobe; BLL, both lower lobe included right/left lower lobe; SUVmax, Maximum standardised uptake value on positron emission tomography/computed tomography scan.

### Diagnostic yield of bronchial brushing or washing

**Figure 1:**
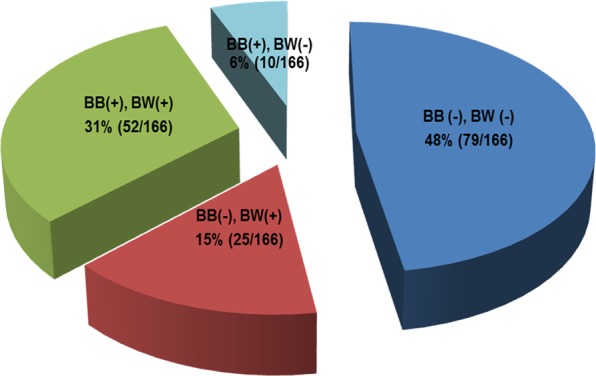
A summary of the diagnostic yield of washing after bronchial brushing in patients with suspected peripheral lung cancer. BB, bronchial brushing; BW, bronchial washing; + malignancy of cytology; −, negative of cytology.

The overall diagnostic yield of bronchial brushing and washing for peripheral lung cancer was 52.4%. The diagnostic yields of brushing and washing were 37.3% and 46.4%, respectively, and that of washing was superior according to McNemar’s test (*p* = 0.017, κ = 0.570). Furthermore, washing was diagnostic, whereas brushing was not, in 15.1% of all cases. Figure 1 summarizes the diagnostic yields of each procedure. Comparison of positive washing cytology (brushing) with the respective pathological diagnosis yielded a concordance rate of 88.3% (90.3%), with κ = 0.769 (0.801) (*p* < 0.001) (Table 2).

**Table 2:** Comparison of the positive cytology results for washing (brushing) with the patient-matched pathology results.

Matched samples	Bronchial washing (brushing) cytology samples (n)				
	ADC	SQC	NSCC	SCC	Total
Adenocarcinoma (ADC)	49 (41)	0 (0)	3 (2)	0 (0)	52 (43)
Squamous cell carcinoma (SQC)	0 (0)	16 (12)	4 (3)	0 (0)	20 (15)
Non-small cell carcinoma (NSCC)	0 (0)	1 (0)	0 (0)	0 (0)	1 (0)
Small cell carcinoma (SCC)	0 (0)	1 (1)	0 (0)	3 (3)	4 (4)
Total	49 (41)	18 (13)	7 (5)	3 (3)	77 (62)

Table 1 shows the factors associated with the diagnostic yield of washing or brushing. In univariate analyses, tumour size, presence of a bronchus sign, and TNM stage were significantly associated with the diagnostic yields of washing and brushing. SUV_max_ on PET/CT was significantly associated with the diagnostic yield of washing, but not brushing. Multivariate analysis showed that a significantly higher diagnostic yield of washing was associated with the bronchus sign only (*p* = 0.001, odds ratio 3.536, 95% confidence interval 1.689–7.400).

The post-bronchoscopic sputum cytology was positive in 37 (26.2%) of the 141 patients from whom specimens were obtained 12–24 hours after FB, and all of these patients were diagnosed by brushing or washing. Of the 106 patients who underwent CT-TTNB, a pathological diagnosis was obtained in 102 (96%). Three of the remaining four patients with negative results had positive cytological results from the brushing and washing samples.

### Complications of bronchoscopy or CT-guided transthoracic needle biopsy

No procedure-related deaths or immediate major complications, such as tension pneumothorax or massive bleeding, occurred with any FB diagnostic procedure. Minor complications after FB included fever ( ≥ 38 °C) in 13 patients and haemoptysis in 8. These conditions resolved spontaneously or by administration of acetaminophen or hydration within 48 hours. Complications of CT-TTNB were assessed by a final CT scan acquired after the procedure. Pneumothorax (*n* = 38) or pulmonary haemorrhage (*n* = 23) developed in 58 (55%) of the 106 patients. Of the 38 patients with pneumothorax, 5 required closed chest tube drainage. Complications in the remaining patients were resolved completely within 48 hours by oxygen therapy (via nasal prongs or a face mask) and/or hydration.

## Discussion

In this study, the overall diagnostic yield of performing washing after brushing in patients with peripheral lung cancer during non-guided FB was 52.4%, and washing had a significantly higher diagnostic yield than that of brushing. Furthermore, 15.1% of all cases had positive washing, but negative brushing, results. These results provide evidence for the optimal sequence of bronchial brushing and washing for the diagnosis of peripheral lung cancer during non-guided FB.

The global sensitivities of brushing and washing for the diagnosis of peripheral lung cancer are 54% (range 16–84%) and 43% (range 12–65%), respectively^3^. However, most previous studies used fluoroscopic guidance routinely, together with TBB. The use of fluoroscopy involves longer procedure times and radiation exposure to both patients and the bronchoscopy team; furthermore, the equipment used for fluoroscopy is often expensive, requires a larger workspace, and is not available universally^4,8–10^. Moreover, the risk of pneumothorax is significantly higher after TBB (1–6%) than after other bronchoscopic procedures (0.1–0.16%)^1^.

Using published data^3^, we reviewed only the reported results for the final diagnosis of peripheral lung cancer during non-guided FB up to 2018 (Table 3). Of nine studies, washing was performed together with TBB in five^8,11–14^, before TBB or brushing in three^12,13,15^, and after other procedures in two studies^8,11^. The average diagnostic yields of brushing and washing from the literature review were 19.6% (range 12.6–31.4%) and 24.4% (range 5.9–50.9%), respectively. Even though most of the previous studies additionally performed TBB, all of our diagnostic yields were higher than those from the literature review (Table 3). Furthermore, the diagnostic yield of washing was more than twice the average diagnostic yield obtained in previous studies (46.4% *vs*. 24.4%). No pneumothorax related to the bronchoscopic procedures was observed in our study. Therefore, washing after brushing during non-guided FB is a very safe diagnostic procedure and may help improve the diagnostic yield in patients with suspected peripheral lung cancer. A prospective study of the timing of bronchial washing for diagnosing lung cancer showed that the diagnostic yields of washing before versus after biopsy and brushing did not differ significantly^16^. For invisible endobronchial tumours, however, the diagnostic yield of washing after brushing and biopsy (42%) tended to exceed that (36%) of washing before brushing and biopsy. This supports our finding that washing after brushing had a better diagnostic yield.

**Table 3:** Diagnostic yields of non-guided flexible bronchoscopic procedures for the final peripheral lung cancer diagnosis based on a literature review.

First author	Year	No.	Order	Diagnostic yield % (positive/cases)			
				Overall	BB	BW or BAL	TBB
Kvale^14^	1976	18	Not mentioned	Not mentioned	27.8 (5/18)	5.9 (1/17)	30 (3/10)
Mak^13^	1990	63	BW,TBB,BB	55.6 (35/63)	28.6 (18/63)	38.1 (24/63)	36.5 (23/63)
Wongsurakiat^12^	1998	30	BAL,TBB	50 (15/30)		46.7 (14/30)	16.7 (5/30)
Rhee^8^	2010	38	BB,TBB,BW	68.4 (26/38)	31.4 (11/35)	32.4 (12/37)	71 (22/31)
Kang^11^	2017	87	BB,TBB,BW	43.7 (38/87)	12.6 (11/87)	3.4 (3/87)	40.2 (35/87)
Pilotti^26^	1982	84	BB	28.6 (24/84)	28.6 (24/84)		
de Gracia^27^	1993	55	BW,BAL,BA	50.9 (28/55)		50.9 (28/55)*	
Sing^28^	1997	145	BB	21.9 (14/64)	21.9 (14/64)		
Labbe^15^	2015	207	BW,BB,BAL	25.6 (53/207)	16.4 (34/207)	18.8 (39/207)*	
**Average form literature**				**38.4** **(209/544)**	**19.6** **(93/474)**	**24.4** **(121/496)**	**39.8** **(88/221)**
**Lim (present study)**	**2019**	**166**	**BB,BW**	**52.4** **(87/166)**	**37.3** **(62/166)**	**46.4** **(77/166)**	

*The diagnostic yield of washings included the results of bronchoalveolar lavage or bronchial aspirated.

BW, Bronchial washing; BB, bronchial brushing; BA, bronchial aspiration; TBB, transbronchial biopsy, BAL, bronchoalveolar lavage;

Now, advanced bronchoscopic techniques include the use of electromagnetic navigation bronchoscopy, virtual bronchoscopy, and an ultrathin bronchoscope combined with radial-probe EBUS, which allows access to peripheral lung lesions not accessible via FB. Generally, FB is routinely performed in the same bronchoscopy session to clear secretions and exclude endobronchial lesions before these advanced bronchoscopy techniques. A meta-analysis showed that the pooled diagnostic yield of advanced bronchoscopy techniques was 70% (range 45–86.2%) across the included studies^17^. However, a recent prospective multicentre study showed that the overall diagnostic yield (53.7%) of advanced bronchoscopy techniques was lower than anticipated. Furthermore, these advanced bronchoscopy techniques are still not routinely available in many centres and are very expensive^6^. Therefore, bronchial brushing and washing are the safest and most cost-effective universal diagnostic techniques for all patients undergoing bronchoscopy. The diagnostic yield of these procedures for diagnosing lung cancer is relatively low, but the use of these procedures is still an essential step in the cost-effective diagnostic workup of patients with suspected lung cancer^16^. We observed an improvement in the diagnostic yield with negative CT-TTNB samples in the cytological results from brushing or washing samples. Although further studies are required to generalise our results, this information will benefit clinicians when performing diagnostic bronchoscopy in patients with suspected peripheral lung cancer in most institutions that cannot offer advanced bronchoscopy techniques. Especially, grafting our results onto the routine FB before advanced bronchoscopy techniques may help improve the diagnostic yield in patients with suspected peripheral lung cancer.

The incredible advancement in liquid biopsy, which refers to the detection and characterisation of molecular tumour profiles by examining circulating tumour cells (CTCs) in blood, may facilitate obtaining an early diagnosis and prognostic or predictive information. Liquid biopsy may also be used to monitor the treatment response, quantify minimal amounts of residual disease, and determine genetic profiles in cancer patients. However, the limited number of CTCs isolated from blood samples requires highly sensitive assays, which are expensive and associated with technical issues. Several studies have detected CTCs in other body fluids, such as urine, saliva, pleural effusions, cerebrospinal fluid, and bronchial samples^18–20^. Sampling of body fluids that directly drain tumor sites may yield higher quantities of CTCs of tumor origin than peripheral blood or plasma^19,21^. A study of molecular mutations detected in bronchial washing or brushing samples obtained by liquid biopsy showed that the detection rate is higher for positive than negative cytological specimens^22^. These results suggest that an accurate cytological diagnosis is important when using bronchial washing samples obtained by liquid biopsy for molecular analyses. Although larger studies are needed to validate this diagnostic approach, our results may provide a foundation for the optimal sequence of washing and brushing for liquid biopsy. Especially, we observed strong agreement between the positive cytology results from washing samples and the respective pathological diagnosis (κ = 0.769).

This study has several limitations and strengths. First, it was a single-institution study without a control group. To overcome this limitation, we compared our diagnostic yields with published data. Although the previous studies reviewed performed TBB in addition to FB, we obtained a higher diagnostic yield than that of those studies (Table 3). Another limitation was the retrospective nature of this study. However, the FB procedures were performed consistently by the same physician using the same protocol with prospective data collection, as mentioned in the Methods. It is possible that the diagnostic yield might by influenced by the expertise of different physicians. All patients in our study had a confirmed pathological diagnosis of lung cancer obtained by procedures other than FB cytology. We believe that this stringent inclusion criterion increased the quality of our study. Despite these limitations, this study provides information about the optimal sequence of bronchial brushing and washing for diagnosing patients with suspected peripheral lung cancer during non-guided FB.

In conclusion, performing washing after brushing during non-guided FB is a very safe, cost-effective procedure that may help improve the diagnostic yield and avoid additional invasive or expensive procedures in patients with suspected peripheral lung cancer. This result may serve as the cornerstone of further studies using bronchial washing samples obtained by liquid biopsy. Our information will also benefit clinicians performing diagnostic bronchoscopy in patients with suspected peripheral lung cancer when fluoroscopic guidance or advanced bronchoscopy techniques are not available.

## Methods

### Study design and patients

Patients with peripheral lung tumours who underwent non-guided FB at Inha University Hospital were enrolled. Since 2010, we have obtained washing samples after brushing the target tumour in these patients. Invisible endobronchial tumours were defined as a normal bronchial system or bronchial narrowing due to extrinsic compression with normal mucosa. Before the FB procedure, we reviewed chest computed tomography (CT) findings, including the target tumour location and size and the presence of a bronchus sign (a finding in the bronchus leading to or contained within the target tumour)^23^. Eligibility criteria were (i) availability of a standard chest radiograph and chest CT before FB, (ii) presence of an invisible endobronchial tumour during non-guided FB, (iii) a washing sample obtained after brushing in a subsegmental bronchus of the target tumour; and (iv) a definite pathological diagnosis of lung cancer established by any diagnostic procedure other than bronchoscopy. Patients diagnosed with other cancers within the previous 5 years were excluded from the study. The patients’ cancers were staged according to the 8^th^ edition of the TNM classification system^24^. The maximum standardised uptake value (SUV_max_) on positron emission tomography (PET)/CT in the target tumour was calculated by a nuclear medicine physician. We retrospectively reviewed the medical records of all consecutive patients meeting the eligibility criteria by 2015. This study was approved by the Institutional Review Board of Inha University Hospital (INHAUH 201809005001). Written informed consent was obtained from all patients before they underwent the FB procedure. All methods were performed in accordance with the relevant guidelines and regulations.

### Bronchoscopy procedures

The same pulmonologist with extensive bronchoscopy experience performed all procedures via the transnasal or transoral route using a variety of video bronchoscopes (models BF-1T260, F260, and 6C260; Olympus; Tokyo, Japan) under local anaesthesia (2% lidocaine spray) and mild conscious sedation with midazolam. Additional doses of lidocaine and midazolam were added during the procedures at the physician’s discretion. After visualising the vocal cords, the trachea and all bronchial trees, including the subsegmental bronchi, were inspected. Bronchial brushing was performed without fluoroscopic guidance using disposable cytology brushes with a covered sheath (BC-202D-2010, Olympus). The brush was passed through the working channel of the bronchoscope while in its sheath and advanced as far as possible into the subsegmental bronchus that was considered to contain the target tumour based on chest CT findings. At this point, the brush and sheath were retracted 3–5 cm, and then the brush was pushed out of the covered sheath and moved back and forth over the lesion several times. After removing the brush from the working channel, 10–20 mL sterile saline were instilled through the working channel in a wedged position in the involved segment, and the washing samples were aspirated. This procedure was repeated two or three times.

### Specimen preparation

Each specimen obtained by bronchial brushing was smeared on two or three clean slides, which were then immediately fixed in 95% ethyl alcohol. All slides were stained with Papanicolaou stain. The bronchial washing samples were subjected to cytological evaluation. The washing samples used for cytological examination were centrifuged for 5 minutes at 2000 rpm and preserved in 95% ethyl alcohol. Within the specimens, cell concentrates were stained with Papanicolaou stain and cellblock sections with haematoxylin and eosin stain following the standard protocol of the pathology laboratory. All slides were interpreted by the same cytopathologist, who was blinded to the patients’ clinical information. Cytological results that clearly indicated a diagnosis of lung cancer were classified as positive, whereas non-diagnostic results or benign, atypical, or suspicious cells without a definitive diagnosis were classified as negative.

### Statistical analysis

The distribution of clinical variables according to brushing or washing cytology was assessed using the chi-square test. The statistical difference in the diagnostic yield of lung cancer between brushing and washing was determined using McNemar’s test. Intra-individual agreement between positive brushing or washing cytology and the final pathological diagnosis was determined by calculating Cohen’s kappa^25^. All analyses were performed using SPSS ver. 19.0 (SPSS, Chicago, IL, USA). Statistical significance was defined as a two-sided *p* < 0.05.

## Data Availability

The dataset supporting the conclusions of the current study is included within the article. The raw data generated and analyzed during the current study are not publicly available since they contain potentially identifying information. However, some raw datasets of the current study are available from the corresponding author on reasonable request.
